# Salvage and storage of infectious disease protein targets in the SSGCID high-throughput crystallization pathway using microfluidics

**DOI:** 10.1107/S1744309111023232

**Published:** 2011-08-13

**Authors:** Jeff Christensen, Cory J. Gerdts, Mathew C. Clifton, Lance Stewart

**Affiliations:** a Emerald BioStructures, 7869 NE Day Road West, Bainbridge Island, WA 98110, USA; b Emerald BioSystems, 7869 NE Day Road West, Bainbridge Island, WA 98110, USA

**Keywords:** microfluidics, salvage, storage

## Abstract

The MPCS Plug Maker is a microcapillary-based protein-crystallization system for generating diffraction-ready crystals from nanovolumes of protein. Crystallization screening using the Plug Maker was used as a salvage pathway for proteins that failed to crystallize during the initial observation period using the traditional sitting-drop vapor-diffusion method. Furthermore, the CrystalCards used to store the crystallization experiments set up by the Plug Maker are shown be a viable container for long-term storage of protein crystals without a discernable loss of diffraction quality with time. Use of the Plug Maker with SSGCID proteins is demonstrated to be an effective crystal-salvage and storage method.

## Abstract 2

SSGCID protein crystals were salvaged and stored using the MPCS Plug Maker and CrystalCards when high-throughput traditional sitting-drop vapor diffusion initially failed.

## 1. Salvaging protein targets

**Figure 1:**
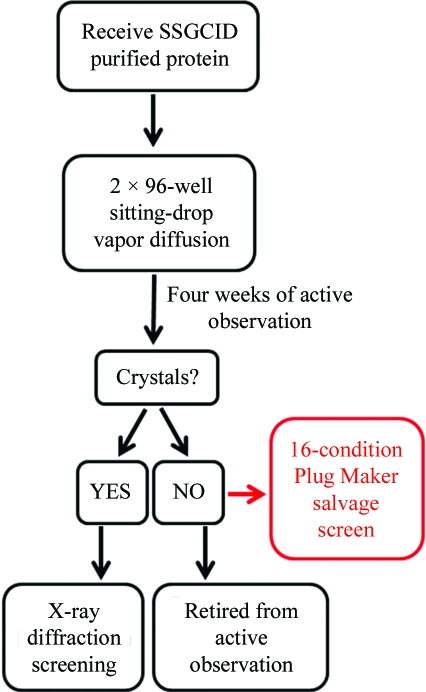
Workflow schematic for the salvage study.

The Seattle Structural Genomics Center for Infectious Disease (SSGCID) seeks to determine the molecular structures of bacterial proteins thought to be good targets for drug therapies, with a target of 75 structures per year over five years. These protein targets arrive at the crystallization core facility at Emerald BioStructures in highly purified and concentrated form as frozen aliquots. A schematic of the workflow used in this study is shown in Fig. 1 ▶. 10–15 new targets arrive at the facility each week and are immediately screened for crystallization using traditional sitting-drop vapor diffusion. Following the strategy proposed by Newman *et al.* (2005 ▶), all targets are screened against two 96-condition screens, JCSG+ (Emerald BioSystems) and PACT Premier (Molecular Dimensions), giving 192 unique conditions. The experiments are monitored for four weeks and any crystals that form enter an optimization pathway that includes testing for protein crystal diffraction, subsequent crystal optimization and structure determination. Inevitably, there are protein targets that produce no crystals within four weeks or fail in the optimization pathways. Typically, these targets are retired from active observation to open space for the next set of incoming targets. Within the Emerald BioStructures crystallization core facility, strategies have been utilized to attempt to salvage the structure determination of retired proteins. Such salvage pathways have included limited proteolysis (McPherson *et al.*, 2004 ▶) and exogenous nucleation (D’Arcy *et al.*, 2003 ▶; Thakur *et al.*, 2007 ▶), although both had very limited success for the required effort involved.

**Figure 2:**
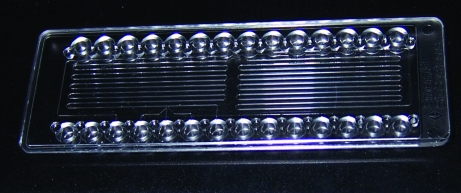
Image of a CrystalCard showing the two microcapillary channels.

With close to 900 retired protein targets currently available for salvage, the MPCS (Microcapillary Protein Crystallization System) Plug Maker (Emerald BioSystems) was used to thoroughly re-screen a small set of retired proteins. The plug-based microfluidic technology encompassed in the Plug Maker is able to thoroughly screen crystallization conditions by quickly and automatically generating con­centration gradients for each condition that is screened. Details of the Plug Maker and its underlying technology have been published previously (Gerdts *et al.*, 2008 ▶, 2010 ▶; Li *et al.*, 2006 ▶; Zheng *et al.*, 2003 ▶, 2005 ▶). Using this technology, as many as 800 20 nl crystallization experiments can be set up in one CrystalCard (Emerald BioSystems; Fig. 2 ▶) using as little as 5 µl protein sample. Furthermore, although they only use up ∼10 nl of protein each, the experiments are large enough to produce diffraction-ready crystals (50–200 µm per side) that can be examined *in situ* or extracted from the peel-apart CrystalCard (Gerdts *et al.*, 2008 ▶, 2010 ▶; Yadav *et al.*, 2005 ▶).

The JCSG+ screen used for the initial trials set up for SSGCID targets arose from data mining conducted by the Joint Center for Structural Genomics, which identified 96 conditions in commercial screens that had been most successful in crystallizing proteins (Newman *et al.*, 2005 ▶; Page & Stevens, 2004 ▶). 16 of these conditions were taken from Wizard Screens I and II (Emerald BioSystems) in order to compose the MPCS Sweet 16 Screen (Table 1 ▶).

Protein targets to be salvaged were screened against all or a portion of the MPCS Sweet 16 Screen based on the availability of remaining protein. No special criteria were used to select the targets other than that they were listed in our tracking spreadsheet as having failed to crystallize in either of the two standard vapor-diffusion trials during the initial four-week screening period. Subsequent to their selection for MPCS screening, the retired targets were re-examined in their original vapor-diffusion plates or selected for additional vapor-diffusion trials for other reasons and ended up crystallizing sometime between two and six months after initial screening. These samples were still included in this analysis because the comparison to the initial screening for retired targets remains valid (Table 3). One of the protein samples was not set up in vapor-diffusion trials because the volume delivered was inadequate and thus went directly to MPCS.

**Figure 3:**
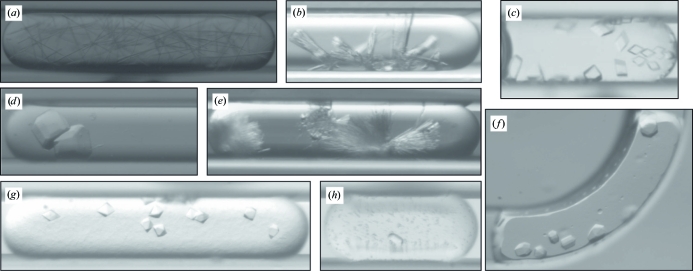
Pictures of crystals obtained in batch-under-oil-style microfluidic experiments in the CrystalCards. The microcapillary width in all pictures is 200 µm. (*a*) MyavA01582cA1; (*b*) MyavA01582aA1; (*c*) MyavA01566cA1; (*d*) MyavA00213aA1; (e) MysmA01566cA1; (*f*) MyavA01582cA1; (*g*) MythA00746aA1; (*h*) GilaA01434aA1

Crystallization experiments were set up in CrystalCards using the Plug Maker instrument. These experiments are analogous to batch crystallization, in which the crystallization reagents are combined in a drop (plug) surrounded by an oil and this initial condition is not expected to change significantly over time (see crystal pictures in Fig. 3 ▶). In this system, each plug is separated by a small volume of inert perfluorinated oil (Fluorinert FC-40, Sigma–Aldrich). The CrystalCard contains two separate microcapillaries and a thorough gradient of as many as 12 different crystallization conditions can be screened in each microcapillary, covering a wide range of crystallization phase space.

## 2. Storing protein crystals

**Figure 4:**
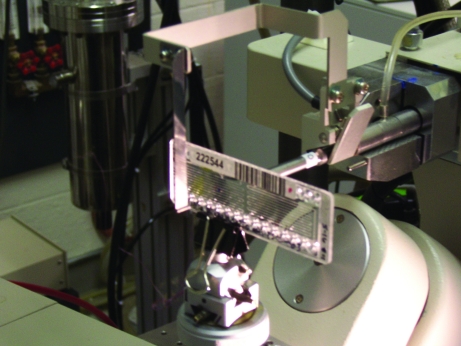
Picture of a CrystalCard mounted on a goniometer for *in situ* diffraction experiments.

**Figure 5:**
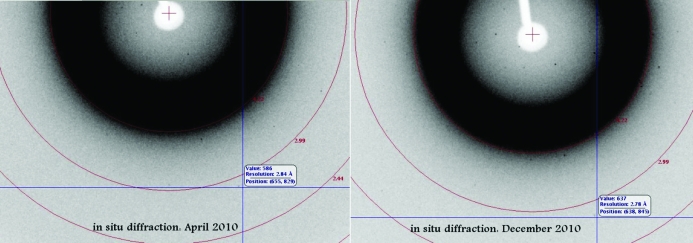
Comparison of *in situ* diffraction of BrabA.00113a.A1 cystals after long-term storage. Diffuse X-ray scatter is a result of the plastic material of the CrystalCard.

Long-term storage of protein crystals at room temperature may be useful for the production of protein crystals for future research (ligand soaking) and may be useful owing to a lack of immediate access to X-ray data collection. Using traditional sitting-drop vapor diffusion or batch-under-oil techniques, protein-crystal storage can be difficult owing to evaporation of the crystallization drop. A long-term study of crystal stability in CrystalCards has shown that crystallization experiments inside CrystalCards can be stored without measurable evaporation and without meaningful loss of protein crystal diffraction quality. Crystals of BrabA.00113a.A1, an infectious disease target from SSCGID, were grown at the same time inside six CrystalCards and were subsequently examined using *in situ* X-ray diffraction by mounting the CrystalCard on the goniometer (Fig. 4 ▶; Gerdts *et al.*, 2008 ▶; Yadav *et al.*, 2005 ▶) periodically from April to December 2010. While not quantified, the crystals appeared to be unchanged and showed no obvious degradation of protein crystal diffraction quality over the course of eight months (Fig. 5 ▶). Furthermore, a 2.6 Å resolution data set was collected from one of the protein targets salvaged in this study (MysmA00391bA1/3C) after the crystals had been stored in the CrystalCard for approximately five months (Table 2 ▶).

## 3. Materials and methods

### 3.1. Crystallization screening conditions

The similarity of microcapillary crystallization to batch crystallization makes it advantageous to start with crystallization solutions at higher component concentrations than are found in commercial sparse-matrix screens; thus, when running a concentration gradient of a crystallization condition against a target protein sample the resulting plugs in the gradient will cover a wider crystallization space. During development of the MPCS Plug Maker system, custom versions of Wizard Screens I and II were designed in which the same reagents were used but at proportionally higher concentrations. Using the 16 conditions common to both the Wizard and JCSG+ screens would allow us to compare the microcapillary results with those of the initial vapor-diffusion trials; these conditions were dubbed the ‘MPCS Sweet 16 Screen’ (see Table 1 ▶).

### 3.2. MPCS parameters

The protein samples were set up using the Plug Maker hybrid protocol. Gradients of conditions 1–8 and 9–16 of the Sweet 16 Screen were run in channels one and two, respectively. Approximately 5 µl protein sample was required to screen all 16 conditions.

### 3.3. CrystalCard storage

CrystalCards were stored in standard plastic microscope slide boxes (VWR) with a small amount of water added to keep them humidified. The boxes were kept at 289 K.

### 3.4. Diffraction testing

For *in situ* diffraction screening at room temperature, CrystalCards were mounted on the goniometer using a special clip (Fig. 4 ▶). The CrystalCards were oriented with the thin backing of the card facing the detector and the plug containing the crystal was aligned in the beam. Rotation of the card in the ω axis is limited to approximately ±15° from 0° because of interference with the beam stop as well as the increasing thickness of the card that the X-ray beam must penetrate when the card is not perpendicular to the beam. A few 30 s/0.5° test images were collected within this range and examined for protein diffraction. If no obvious diffraction was noted a salt test was performed.

### 3.5. Salt test

With the card mounted and aligned as above, the detector was moved in to 50 mm and the 2θ axis moved out to 45°. A wedge of 1°/10 s images at least 15° wide was collected and if no small-molecule reflections were observed the crystal was characterized as ‘not salt’.

## 4. Results and discussion

Microfluidic salvage efforts were attempted on 34 targets. 23 produced crystals or crystal-like objects. Through *in situ* X-ray diffraction screening eight of these showed obvious protein diffraction patterns, and complete data sets to ∼2.6 Å resolution were collected for two targets from crystals harvested directly from the CrystalCards (Table 2 ▶). Because the crystals screened at room temperature may have sustained radiation damage, the data sets were collected at 100 K using different crystals from the same cards. Of the remaining crystals, 11 were characterized as ‘likely protein’ owing to a few very low-resolution reflections close to the beam stop, two were characterized as ‘not salt’ after failing the salt test (see §[Sec sec3]3) and two were confirmed as being salt crystals (Table 3 ▶).

The initial vapor-diffusion screening of SSGCID samples against only two 96-condition screens is minimal and has allowed the crystallization group to achieve the annual goals outlined in the goals of the SSGCID. Screening these same samples against a larger number of sparse-matrix screens may have resulted in crystals for a larger percentage of samples, but would come with the additional burden of more trials to set up, monitor and store, and a greater cost of consumables. Using the capabilities of the Plug Maker to screen a wide variety of concentrations of just 16 crystallization screening conditions has resulted in two high-resolution data sets and legitimate crystallization leads for many previously retired targets. While all of the vapor-diffusion crystals reported here appeared after four weeks, many of the MPCS crystals appeared in the CrystalCards within days.

The results of these preliminary MPCS experiments are very encouraging. Although nominally using a screen of only 16 conditions, the actual number of unique conditions is the number of plugs in the CrystalCard (up to ∼800) because each crystallization solution is run as a gradient. The ability to obtain crystallization leads, protein crystal diffraction and full diffraction data sets using less than 10 µl protein sample, and with a success rate comparable to standard crystallization methods, represents a large saving in effort and indicates that efficient microfluidic scanning of protein and crystallization condition concentrations in initial crystallization trials using the MPCS Plug Maker can be a highly effective means of producing protein crystals.

In addition to soluble proteins, the Plug Maker may prove to be useful for crystallizing nucleic acids and membrane proteins, both of which represent special challenges. While preliminary experiments have shown that membrane proteins solubilized with some detergents form plugs normally, neither nucleic acids nor membrane proteins have been tested extensively by us.  Experiments are planned to more thoroughly investigate this and the results of these experiments will be reported in due course.

## Figures and Tables

**Table 1:** Concentrated conditions composing the MPCS screen (MPCS Sweet 16)

MPCS Screen No.	Wizard Full No.	JCSG+		At 100 m*M*	At 200 m*M*
1	1	A-7	30%(*w*/*v*) PEG 8000	CHES pH 9.5	None
2	2	E-3	20%(*v*/*v*) 2-propanol	HEPES pH 7.5	NaCl
3	6	A-2	30%(*w*/*v*) PEG 3000	Citrate pH 5.5	None
4	9	E-8	1.5 *M* (NH_4_)_2_HPO_4_	Acetate pH 4.5	None
5	14	E-1	1.4 *M* sodium citrate	Cacodylate pH 6.5	None
6	17	D-4	40%(*w*/*v*) PEG 8000	Acetate pH 4.5	Li_2_SO_4_
7	31	C-1	30%(*w*/*v*) PEG 8000	Phosphate–citrate pH 4.2	NaCl
8	39	A-6	30%(*w*/*v*) PEG 1000	Phosphate–citrate pH 4.2	Li_2_SO_4_
9	47	E-4	2.52 *M* (NH_4_)_2_SO_4_	Tris pH 8.5	Li_2_SO_4_
10	49	C-7	20%(*w*/*v*) PEG 3000	Acetate pH 4.5	Zn(OAc)_2_
11	51	D-6	30%(*w*/*v*) PEG 8000	Tris pH 8.5	MgCl_2_
12	52	E-2	3.0 *M* (NH_4_)_2_SO_4_	Cacodylate pH 6.5	NaCl
13	59	E-7	20%(*v*/*v*) 2-propanol	Cacodylate pH 6.5	Zn(OAc)_2_
14	82	E-12	20%(*w*/*v*) PEG 8000	Imidazole pH 8.0	None
15	88	E-6	30%(*w*/*v*) PEG 3000	Imidazole pH 8.0	Zn(OAc)_2_
16	91	B-8	20%(*w*/*v*) PEG 8000	Tris pH 7.0	MgCl_2_

**Table 2:** Data-collection statistics Values in parentheses are for the highest of 20 resolution shells

	MysmA.00391.bA1/3C	MysmA.01566.cA1
Space group	*P*2_1_	*R*3/*R*3_2_
Unit-cell parameters (Å, °)	*a* = 57.6, *b* = 95.6, *c* = 115.3, β = 99.6	*a* = *b* = 87.8, *c* = 183.9
Wavelength (Å)	1.5418	1.5418
Resolution range (Å)	50–2.8 (2.90–2.80)	50–2.6 (2.69–2.60)
No. of unique reflections	30844 (3076)	16285 (1623)
Multiplicity	3.5 (3.4)	2.7 (2.7)
Completeness	99.6 (100)	99.6 (99.9)
*R*_merge_†	0.13 (0.48)	0.13 (0.49)
Mean *I*/σ(*I*)	6.94 (2.66)	6.87 (2.2)

† *R*_merge_ = 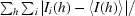 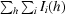.

**Table 3:** Summary of MPCS crystallization results

			MPCS CrystalCard *in situ* diffraction screening and data collection				
SSGCID target protein	MPCS sample No.	MPCS crystals†	Protein diffraction‡	Likely protein	Not salt	Salt	Vapor-diffusion crystals in retired trays§
AnphA.00481.aA1	1						
AnphA.00462.aA1	2						
BaheA.00227.aA1	3						
BaheA.01161.aA1/3C	4						
EnhiA.01533.aA1	5	*		X			
GilaA.01434.aA1	6	*		X			
LedoA.00425.aAG11	7	*			*		*
MyavA.00213.aA1	8	*	XX				*
MyavA.00754.aA1	9	*	XX				
MyavA.00814.aA1	10						
MyavA.00937.aA1	11	*		X			*
MyavA.01097.hA1	12						
MyavA.01097.iA1	13	*			*		*
MyavA.01188.aA1	14	*		X			
MyavA.01212.aA1	15	*	XX				*
MyavA.01326.lA1	16						
MyavA.01379.aA1	17	*		X			*
MyavA.01530.cA1	18	*		X			*
MyavA.01549.bA1	19						
MyavA.01582.aA1	20	*	XX				PDB 3qlj
MyavA.01582.cA1	21	*	XX				*
MyavA.01625.aA1	22	*		X			*
MyavA.01649.cA1	23	*		X			
MyavA.10520.bA1	24	*		X			
MyavA.17065.aA1	25						
MyleA.00778.aA1	26	*		X			*
MyleA.01155.aA1	27						
MypaA.00249.aA1	28						
MysmA.00337.bA1	29	*				*	
MysmA.00391.bA1/3C	30	*	XXX				*
MysmA.01263.bA1	31	*				*	
MysmA.01566.bA1	32	*		X			
MysmA.01566.cA1	33	*	XXX				*
MythA.00746.aA1	34	*	XX				*
Totals	34	23	8	11	2	2	14

† An asterisk indicates the presence of crystals.

‡ X, evidence of protein crystal diffraction *in situ* in the CrystalCard; XX, clear evidence of protein crystal diffraction *in situ* in the CrystalCard; XXX, complete X-ray diffraction data set collected from a crystal extracted from the peeled-apart CrystalCard.

§ An asterisk indicates the presence of crystals. A structure was deposited for MyavA.01582.aA1 with PDB code 3qlj.
